# Supplementary material for: CoO Nanozymes with Multiple Catalytic Activities Regulate Atopic Dermatitis
Source: Nanomaterials (Basel). 2022 Feb 14;12(4):638. doi: 10.3390/nano12040638 (PMC8878353; doi:10.3390/nano12040638)
Supplement: Supplementary file 1 [file nanomaterials-12-00638-s001.zip › nanomaterials-1564340-supplementary.pdf]

Supplementary Materials

CoO Nanozymes with Multiple Catalytic Activities Regulate Atopic Dermatitis

Mao Mao [1,2,†], Xuejiao Guan [1,2,†], Feng Wu [1,2] and Lan Ma [2,3,4,*]

[1] School of Life Sciences, Tsinghua University, Beijing 100084, China; larva1949@126.com (M.M.); gxj920309@126.com (X.G.); wu8721@126.com (F.W.)

[2] Institute of Biopharmaceutical and Health Engineering, Tsinghua Shenzhen International Graduate School, Tsinghua University, Shenzhen 518055, China

[3] State Key Laboratory of Chemical Oncogenomics, Tsinghua Shenzhen International Graduate School, Tsinghua University, Shenzhen 518055, China

[4] Institute of Biomedical Health Technology and Engineering, Shenzhen Bay Laboratory, Shenzhen 518055, China

* Correspondence: malan@sz.tsinghua.edu.cn; Tel./Fax: +86-755-26033033

† These authors contributed equally to this work and should be considered co-first authors.

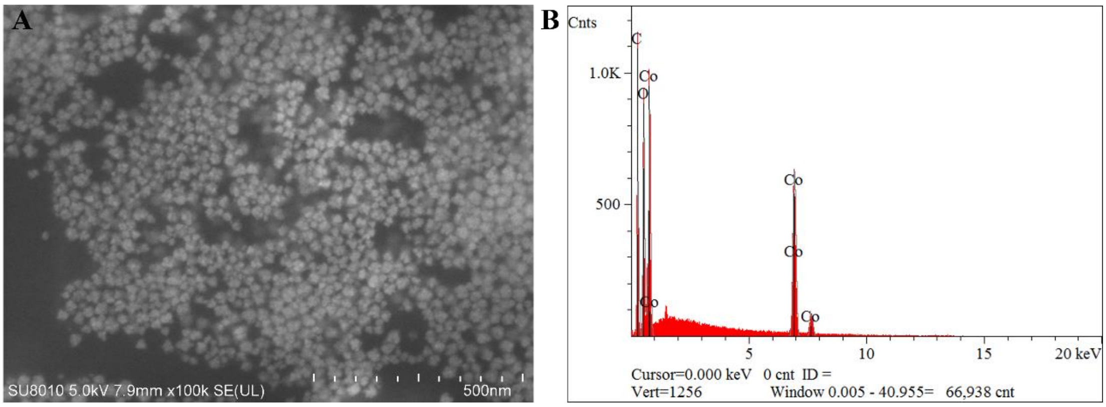

Figure S1. The SEM images and EDS analysis of CoO nanoparticles. (**A**) The SEM image of hydrophobic CoO nanoparticles. (**B**) EDS analysis of CoO nanoparticles.

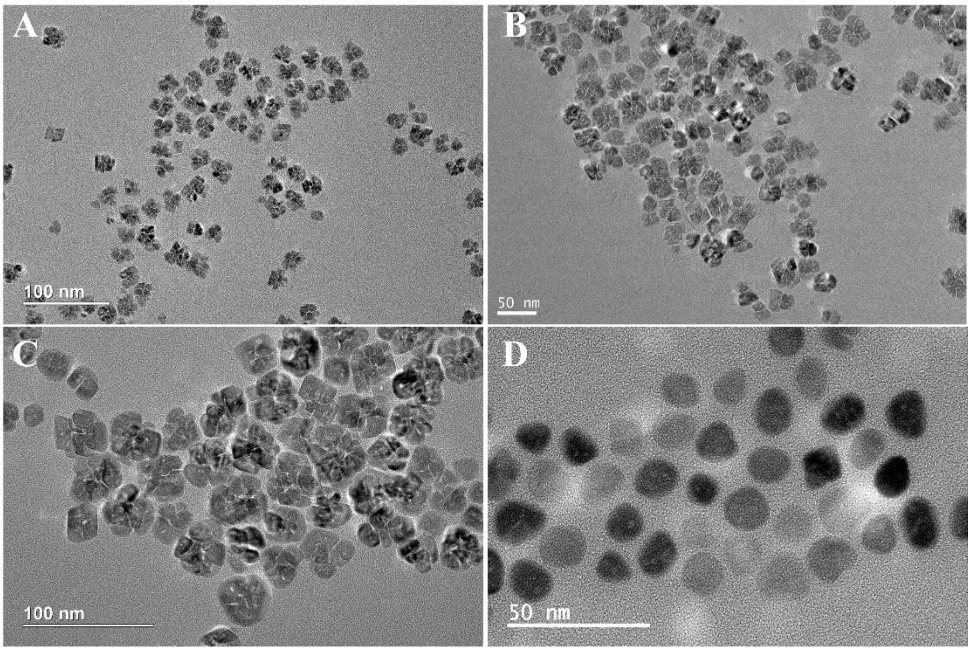

Figure S2. TEM images of CoO nanoparticles synthesized at different temperatures. (**A**) The TEM image of hydrophobic CoO nanoparticles prepared at 240 °C. (**B**) Synthesized at 270 °C. (**C**) Synthesized at 300 °C. (**D**) Synthesized at 320 °C.

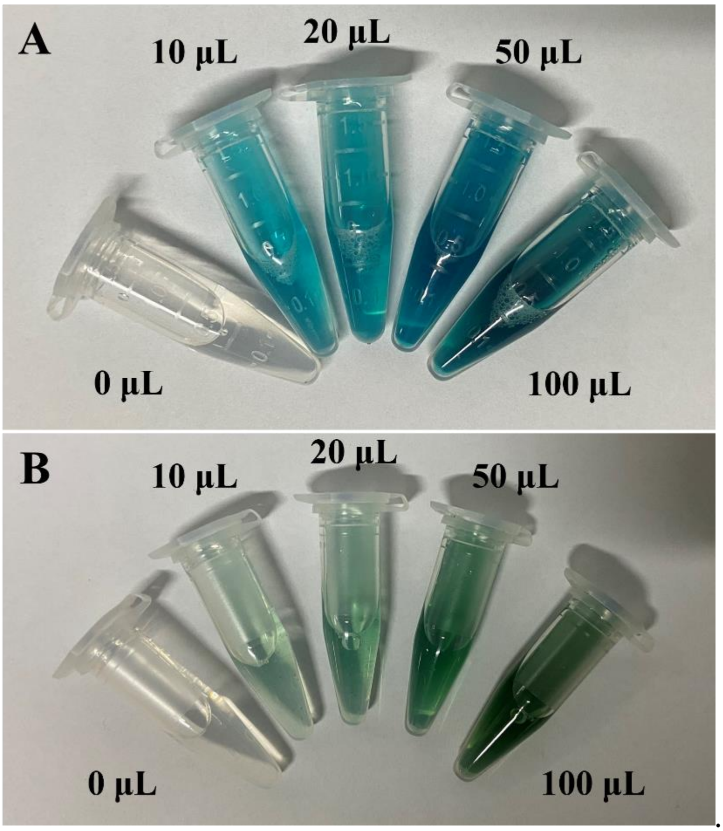

Figure S3. The images of different concentrations of CoO nanozymes reacting with TMB for 10 s. (**A**) Color Reaction of CoO Nanozymes Prepared at 270 °C with TMB. (**B**) Color Reaction of CoO Nanozymes Prepared at 320 °C with TMB.

1. Enzyme-like Activity Determination of CoO Nanozymes

1.1. Determination of SOD-like Catalytic Activity of CoO Nanozyme

The Biyuntian SOD detection kit (WST-8 method) was used to determine the SOD-like activity of CoO NPs at different concentrations (5, 10, 20, and 30 μg/mL).

The Biyuntian SOD detection kit (WST-8 method) is a colorimetric reaction based on WST-8, a kit for detecting SOD or superoxide dismutase activity in cells, tissues or other samples by colorimetry. Refer to Figure S4 for the principle of WST-8 method. WST-8 can react with superoxide anion ($O_2^{\bullet-}$) catalyzed by xanthine oxidase (XO) to produce water-soluble formazan dye. Since SOD can catalyze the disproportionation of superoxide anions, this reaction step can be inhibited by SOD, so the activity of SOD is negatively correlated with the generation of formazan dye. Thus the enzymatic activity of SOD can be calculated by colorimetric analysis of the WST-8 product. The operation steps are as follows:

1) WST-8/enzyme working solution configuration: WST-8/ enzyme working solution was configured according to the volume of each reaction 160L (151 uL SOD detection buffer, 8 μ L WST-8 and 1 μL enzyme solution);

2) Reaction starting solution configuration: Mix 40× reaction starting solution (1 μL) and SOD detection buffer (39 μL) according to the ratio of 1:39 to configure the reaction starting solution;

3) Set sample wells and samples in a 96-well plate. For blank control wells, the reaction system should be in accordance with Table S1. The reaction starting solution should be added at the end.

Table S1. Reaction system for determination of SOD activity.

	**Sample**	**Blank Control 1**	**Blank Control 2**	**Blank Control 3**
CoO nanozymes	20 μl	-	-	20 μl
SOD detection buffer	-	20 μl	40 μl	20 μl
WST/8 enzyme working solution	160 μl	160 μl	160 μl	160 μl
Reaction start-up solution	20 μl	20 μl	-	-

4) Incubate at 37 °C for 30 min, and measure A450;

5) The $O_2^{\bullet-}$ inhibition percentage of CoO Nanozymes = $[(A_{blank\ control\ 1}-A_{blank\ control\ 2})-(A_{sample}-A_{blank\ control\ 3})]/(A_{blank\ control\ 1}-A_{blank\ control\ 2})\times 100\%$.

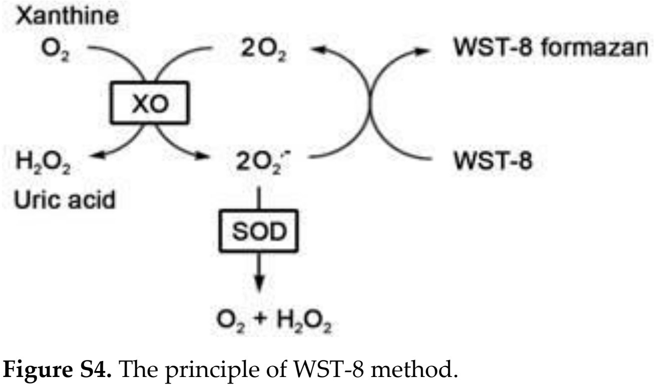

Figure S4. The principle of WST-8 method.

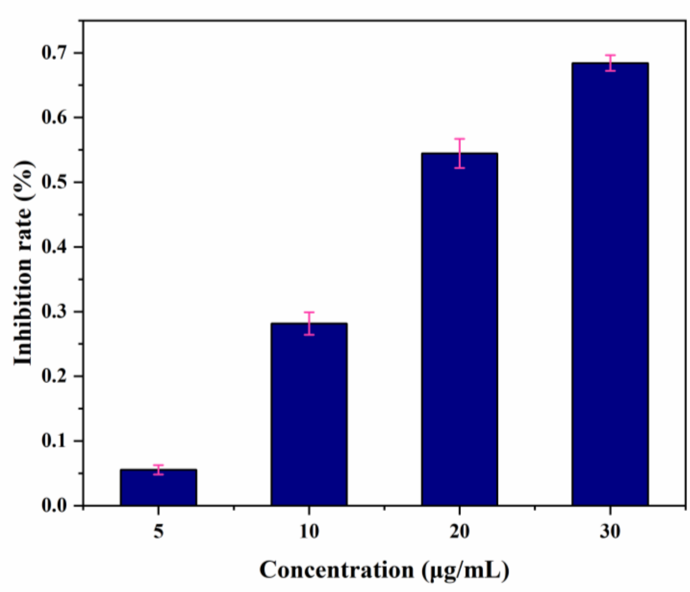

Figure S5. The inhibition rate of $O_2^{\bullet-}$ by different concentrations of CoO nanozymes.

Figure S5 shows the inhibition rate of $O_2^{\bullet-}$ with different concentrations of CoO nanozymes. The inhibition rates of CoO nanozymes with concentrations of 5, 10, 20 and 30 μg/mL to $O_2^{\bullet-}$ were 5.56, 28.15, 54.46 and 68.43%, respectively.

1.2. Determination of CAT-like Catalytic Activity of CoO Nanozyme

The Biyuntian CAT detection kit was used to determine the CAT-like activity of CoO nanozymes at different concentrations (10 and 20 μg/mL).

The Biyuntian CAT detection kit is a simple and easy-to-use kit for detecting catalase (Catalase) activity in cells, tissues or other samples by color reaction. When hydrogen peroxide is relatively abundant, catalase can catalyze the production of water and oxygen from hydrogen peroxide. Residual hydrogen peroxide can oxidize the chromogenic substrate under the catalysis of peroxidase to produce a red product (N-(4-antipyryl)-3-chloro-5-sulfonate-p-benzoquinonemonoimine), and the maximum absorption wavelength is 520 nm. Using the hydrogen peroxide standard, make a standard curve, so that the catalase in the sample can calculate how much hydrogen peroxide catalyzes the conversion of hydrogen peroxide into water and oxygen per unit time and unit volume. Thus, the enzyme activity of catalase in the sample can be calculated. The steps are given below:

1) Determine the standard curve: The H_2O_2 dilution with a final concentration of 0, 0.0025, 0.005, 0.01, 0.015 μM was reacted with catalase, A520 nm was determined and the standard curve was made;

2) CoO nanozymes with different concentrations (10 and 20 μg/mL) were mixed with H_2O_2 detection buffer (total volume of 40 μL), 10 μL of 250 mM H_2O_2 solution was added, and react for 3 min at 25 °C;

3) 450 μL of catalase reaction termination solution were added while mixing upside down to terminate the reaction;

4) Took another 1.5 mL EP tube, added 40 μL of catalase detection buffer, and then added 10 μL of the reaction solution in step 3), and mixed;

5) Adding 10 μL in 50 μL system to 96-well plate in step 4), adding 200 μL chromogenic working solution in each well;

6) Incubated at 25 °C for 15 min, and measured A520 nm;

7) First, the amount of residual H_2O_2 after the reaction of 10 and 20 μ g/mL CoO NPs with H_2O_2 was calculated according to the standard curve, the amount of H_2O_2 consumed is obtained by subtracting the amount of residual H_2O_2 from the amount of residual H_2O_2 of the blank control, and then the CAT-like activity of CoO nanozymes = the amount of consumed H_2O_2 × dilution multiple / (reaction time × sample volume).

A

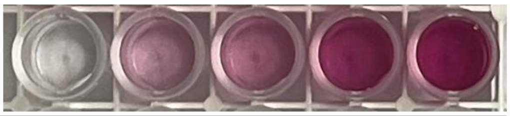

H_2O_2 (μM)	0	0.0025	0.005	0.01	0.015
A520 (a.u.)	0.0486	0.2278	0.3675	0.7824	1.0858

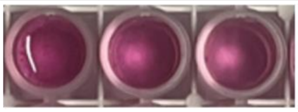

CoO nanozymes (μg/mL)	0	10	20
A520 (a.u.)	0.771	0.681	0.556

B

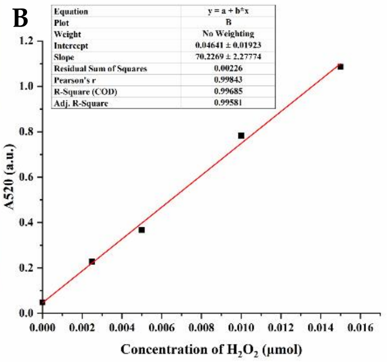

Figure S6. The CAT-like activity assay of CoO nanozymes. (**A**) Test results using the Biyuntian CAT detection kit. (**B**) The standard curve of H_2O_2.

As the concentration of H_2O_2 increases, its absorbance increases linearly. According to the standard curve calculation, CoO nanozymes have CAT-like properties, and their CAT-like activity is about 19.01 U/mL.

1.3. Determination of POD-like Catalytic Activity of CoO Nanozyme

The POD-like activity of CoO nanozyme was determined according to Sigma peroxidase activity assay kit. Peroxidase is an enzyme widely found in biological systems that use hydrogen peroxide to oxidize various substrates. The peroxidase activity assay kit provides a simple and direct procedure for measuring peroxidase activity in various biological samples. Peroxidase catalyzes the reaction between H_2O_2 and the probe, resulting in a colorimetric (570 nm)/fluorescent (λex = 535/λem = 587 nm) product proportional to the peroxidase activity present. One unit of peroxidase is defined as the amount of enzyme that reduces 1.0 μmol of H_2O_2 per min at 37 °C. The following is the operation procedure:

1) Determine the standard curve of H_2O_2: First, dissolve 10 μL of 12.5 mM H_2O_2 in 1240 μL of Assay Buffer, and add 0, 10, 20, 30, 40, and 50 μL to the well plate; and use Assay Buffer to fill each well. To 50 μL. Subsequently, add 2 μL fluorescent substrate and 48 μL HRP positive control to each well, measure A570 nm and make a standard curve;

2) Solution premix (46 μL Assay Buffer, 2 μL fluorescent substrate and 2 μL 12.5 mM H_2O_2), add Mix 50 μL of the master mix with CoO nanozymes. Incubate at 37 °C for 3 min, measure the initial A570 nm, and then measure the A570 nm at this time after 3 min, calculate the amount of H_2O_2 consumed according to the standard curve;

3) Calculate the POD-like activity of CoO nanozymes = the amount of H_2O_2 consumed × dilution Multiple/(reaction time × sample volume).

A

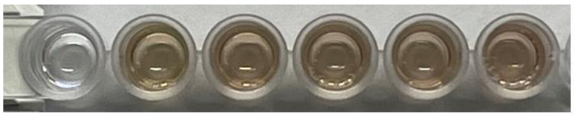

H_2O_2 (nmol)	0	1	2	3	4	5
A570 (a.u.)	0.0474	0.1925	0.3017	0.4925	0.6117	0.7581

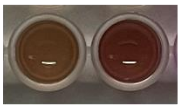

CoO nanozymes (μg/mL)	20	30
Consumption of H_2O_2	0.706	1.051

B

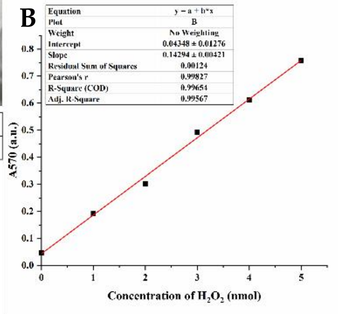

Figure S7. The POD-like activity assay of CoO nanozymes. (**A**) Test results using the Sigma peroxidase activity assay kit. (**B**) The standard curve of H_2O_2.

According to the standard curve, CoO nanozymes have POD-like catalytic activity, and their POD-like activity is about 18.47 U/mL.
